# *The Africa We Want* : A Perspective on the African Code Against Cancer To Be Developed for Sub‐Saharan Africa

**DOI:** 10.1002/ijc.70703

**Published:** 2026-08-27

**Authors:** Anthony Kityo, Phyllis Ohene‐Agyei, Felix M. Onyije, Hannah Simba, Annet Nakaganda, Denis Chopera, Kofi Mensah Nyarko, Benido Impouma, Mary Nyangasi, Blandina Mmbaga, Valerie McCormack, Cesaltina Lorenzoni, Miriam Mutebi, Joachim Schüz, Carolina Espina

**Affiliations:** ^1^ Environment and Lifestyle Epidemiology Branch International Agency for Research on Cancer Lyon France; ^2^ African Population and Health Research Centre Nairobi Kenya; ^3^ Uganda Cancer Institute and Ananda Centre for Cancer Research (ACCR) Kampala Uganda; ^4^ Science for Africa Foundation Nairobi Kenya; ^5^ World Health Organization Regional Office for Africa Brazzaville Congo; ^6^ World Health Organization Headquarters Geneva Switzerland; ^7^ School of Medicine Kilimanjaro Christian Medical Center Tanzania; ^8^ Hospital Central de Maputo Maputo Moçambique; ^9^ Faculdade de Medicina da Universidade Eduardo Mondlane Maputo Moçambique; ^10^ African Organisation for Research and Training in Cancer Cape Town South Africa; ^11^ Department of Surgery Aga Khan University Nairobi Kenya

**Keywords:** cancer control, cancer prevention, National Cancer Control Plans, sub‐Saharan Africa, World Code against cancer framework

## Abstract

Sub‐Saharan Africa (SSA) faces a unique cancer burden, characterized by a disproportionately high mortality‐to‐incidence ratio, a large share of infection‐related cancers, and distinct environmental exposures. Implementation of national policies on primary prevention is limited, and public literacy regarding cancer risk and curability remains low. Furthermore, the implementation of existing prevention and treatment interventions is hindered by resource constraints and deeply rooted cultural beliefs. Thus, cancer prevention guidelines developed for other regions are often inadequate for this specific context. Following the International Agency for Research on Cancer's strategic and methodological World Code Against Cancer Framework—which develops region‐specific, evidence‐based recommendations—we call to join forces for the development of an African Code Against Cancer (AfCAC). The AfCAC would be targeted to the public and policymakers, tailored to the epidemiological, socio‐cultural, and health system realities of SSA, and incorporated into existing National Cancer Control Plans (NCCPs). This Code would be aligned with the most relevant risk factors, local knowledge, and culturally appropriate effective cancer preventive interventions. Successful development of the AfCAC will require sustained commitment from diverse African stakeholders, including national governments, key scientists, non‐governmental actors and funders. Successful implementation will necessitate multistakeholder input such as from policy implementers, advocacy groups, social anthropologists, religious leaders, and traditional healers and other local multisectoral stakeholders who possess a nuanced understanding of local norms integral to cancer prevention efforts. Importantly, strong national governmental commitment, demonstrated through developing and funding effective NCCPs and robust cancer registries, alongside capacity building for local health and non‐health actors, will be paramount.

## Graphical abstract

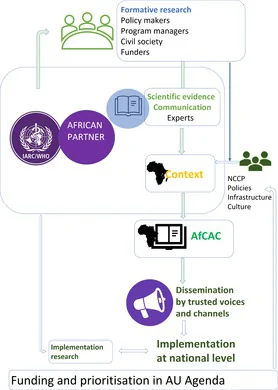

**Abbreviations**
AfCAC: African Code Against Cancer
AfCDC: Africa Centre for Disease Control and Prevention
AFRO: WHO Regional Office for Africa
AORTIC: African Organisation for Research and Training in Cancer
ASR: age standardized rates
AU: African Union
CDs: communicable diseases
DEE: diesel engine exhaust
ECAC: European Code Against Cancer
EU: European Union
GDP: gross domestic product
HCC: hepatocellular carcinoma
HIC: high income countries
HIV: human immunodeficiency virus
HPV: human papilloma virus
IARC: International Agency for Research on Cancer
KS: Kaposi's sarcoma
LAC: Latin America and the Caribbean
NCCPs: National Cancer Control Plans
NCDP: non‐communicable diseases plans
NCDs: non‐communicable diseases
NHL: non‐Hodgkin's lymphoma
PAHs: polycyclic aromatic hydrocarbons
SSA: sub‐Saharan Africa
WCACF: World Codes Against Cancer Framework
WHA: World Health Assembly
WHO: World Health Organization

## 1 Introduction

Sub‐Saharan Africa (SSA) faces a unique double burden of morbidity, characterized by persistent communicable diseases (CDs) and increasing prevalence of non‐communicable diseases (NCDs). By 2030, NCDs will surpass CDs as the leading causes of mortality and disability [1]. Perceived to be rare and a disease of the affluent in the past, cancer is now the second cause of NCD‐related morbidity and mortality in SSA [2]. According to GLOBOCAN estimates from 2022, 847,974 new cancer cases and 558,878 deaths were reported in SSA [3]. These figures are projected to double by 2040 [3] and exceed the global average by 30% [2]. Although the 2022 incidence rate was relatively low, that is, age standardized rate of 132/100000 compared to the global rate of 196/100,000, outcomes or survival levels are disproportionally poor in SSA. Explicitly, the ratio of deaths to incidence is 7–10 in SSA compared to 4–10 in high income countries (HIC) [4].

These stark inequalities reflect systematic and structural barriers to cancer prevention and care, given that a significant proportion of the cancer burden in SSA is preventable [5]. For example, cervical cancer, a rare cause of death in Europe and North America, but the leading cause of cancer mortality among women in 27 African countries [3], is preventable with Human Papilloma Virus (HPV) vaccination and population‐based effective screening strategies [6]. Sadly, only 48% of women in SSA received the full HPV vaccine dose in 2023 [7], while screening rates have been reported at 14% [8]. These figures fall well below the WHO 2030 targets of 90% for vaccination, 70% for screening and 90% for treatment, the three of which are critical to being well on the path to eliminating cervical cancer [8].

In addition to adverse health outcomes, cancer also poses a significant economic burden in Africa, projected at 0.24% of total GDP between 2020 and 2050 and amounting to 395 billion international dollars or $241 per capita in out‐of‐pocket expenditure for cancer treatment [9]. While this is the lowest globally, it represents a significant economic burden in the SSA region with direct costs (healthcare expenditures and disability costs) and indirect costs. Globally, the indirect costs including premature deaths and productivity loss due to loss of income, absenteeism and presenteeism are highest in SSA [10]. Furthermore, SSA has the youngest population globally, and a high prevalence of late stage at presentation and premature mortality among cancer patients. This has created an orphan crisis on the continent, estimated at two orphans per death of a woman under 50 years old [11]. The increasing cancer burden, high case fatality rate, large economic burden and transgenerational effects necessitate a context‐specific approach to cancer prevention in SSA.

Nearly 35% of cancer cases and more than 60% of cancer deaths in SSA are avoidable [5, 12]. The 2017 landmark WHO Cancer Resolution on cancer prevention and control resounded cancer control as a public health priority, and urged governments to promote primary prevention of cancer, including by promoting access to vaccinations to control infection‐related cancers and tobacco use [13]. Although 81% of African countries currently have national cancer control plans (NCCP) that incorporate prevention elements [14], implementation of these programs has been limited because of systemic and social barriers, including limited cancer literacy and cultural beliefs [15]. As a result, population reach of key cancer prevention interventions has been reported at under 50% [16]. Here, we highlight the timely need to develop systematic, evidence‐based recommendations for cancer prevention for SSA, herein the African Code Against Cancer (AfCAC).

## 2 Rationale for an African Code Against Cancer

The World Codes Against Cancer Framework (WCACF) [17], an International Agency for Research on Cancer (IARC)‐led initiative, seeks to provide region‐specific, evidence‐based recommendations for cancer prevention following rigorous scientific methodology. This approach acknowledges that existing cancer prevention guidelines, although partly adaptable, are non‐universal and typically inadequate to address contextual drivers of cancer burdens in different regions [18]. The pioneering European Code Against Cancer (ECAC) are individual‐tailored recommendations on elimination or reduction of exposure to established cancer risk factors prevalent in the European Union (EU), and effective interventions suited to the features of the EU health systems and policy landscape. Adherence to the ECAC, which recently launched its fifth version, could reduce almost half of cancer cases in this region [19].

However, similar benefits may not be reaped in SSA where at present, leading cancers are linked to infections, reproductive factors, and socioeconomic drivers compared to more dominant lifestyle‐related risk factors in Europe [3, 19]. For the same reason of adaptation to local contexts, the first edition of the Latin America and the Caribbean (LAC) Code Against Cancer was developed along with local experts' scientific assessments and review of global and regional evidence, as described in the WCACF. This was the first time a Code was successfully developed for other regions outside of Europe. The LAC Code provides 17 recommendations—not only to individuals, but also policy makers—to structurally shape individual choices and reduce involuntary environmental exposures, and tailored to the unique cancer epidemiological, socio‐economic and health systems' context in LAC [20].

The LAC Code echoes the SSA reality due to a similar profile of the top incident cancers in both regions [3], and a strong emphasis on highly prevalent infection‐related cancers. Following the WCACF methodology, a future AfCAC would take the latest existing Code Against Cancer as starting point to review and update evidence, accounting for the distinct cancer and risk factor profiles that SSA faces, and the differing sociocultural contexts that influence delivery and uptake of cancer prevention interventions. Ultimately, the code would propose the most suitable communication to make evidence‐based recommendations to both individuals and policy makers, acceptable by the African populations and feasible for their health systems. The justifications for the AfCAC development are described in detail in the following sections.

### 2.1 Highest Projected Increase in Cancer Burden

According to 2022 GLOBOCAN estimates, SSA is projected to experience the highest rise in the global cancer burden over the coming decades, driven largely by rapid population growth and ageing (Figure 1). By 2050, the region is projected to account for the largest absolute increase in cancer cases and deaths worldwide, projected to be nearly five times greater than in Europe and double that of other regions [21]. An epidemiological shift is anticipated, with rising incidence of lifestyle‐related cancers such as breast and colorectal cancers, and persistence of infection‐related cancers such as cervical and liver cancer. Notably, absolute numbers of cancers that currently contribute a relatively small share of the regional burden are also projected to rise sharply; for example, gastric cancer—which is historically of low incidence in SSA—is projected to increase substantially, turning the region into a gastric cancer hotspot by 2050 [22].

**FIGURE 1:**
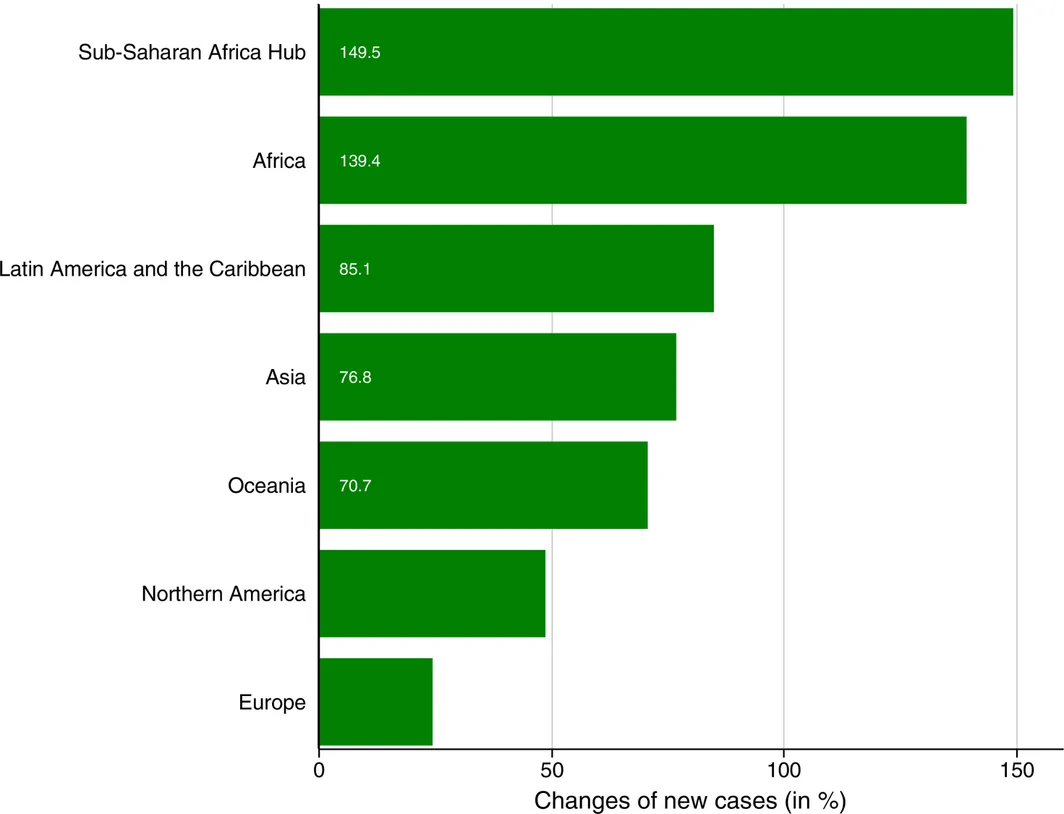
Global changes of new cancers from 2022 to 2045 (as estimated by GLOBOCAN 2022).

Without substantial investment in cancer prevention and control, these trends suggest that SSA will likely be the epicenter of the future global cancer burden.

### 2.2 High Proportion of Cancers Associated With Infections

In Africa, six of the 15 most common cancers are associated with infections (Figure 2), a markedly higher proportion than in LAC and other world regions [23].

**FIGURE 2:**
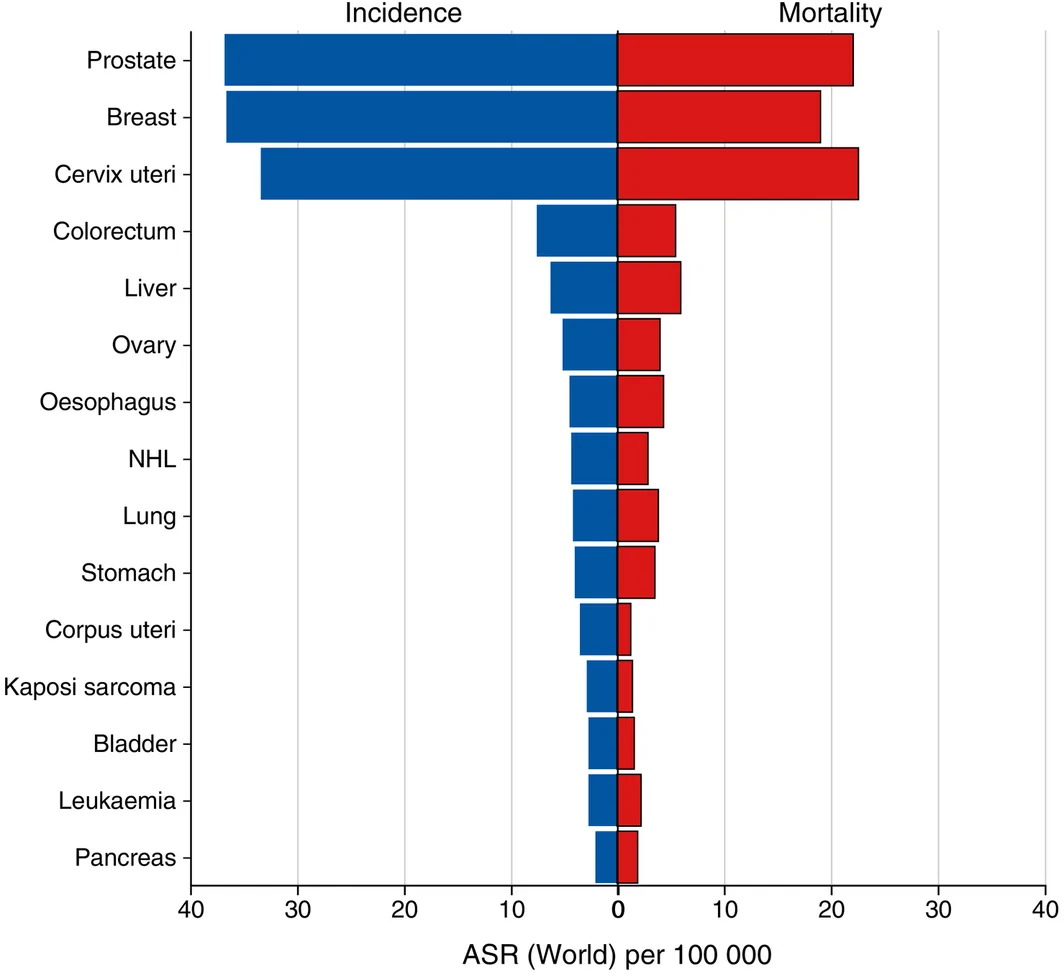
Incidence and mortality of 15 cancers in 2022 in SSA (as estimated by GLOBOCAN 2022). ASR, age standardised rates; NHL, non‐Hodgkins' lymphoma.

Nearly 25% of cancers in Africa are attributable to infections (Figure 3), twice higher than in LAC and more than five times in Western countries [12]. Cervical cancer, for instance, which is the leading cancer among women in Africa, is caused by HPV. Other infection‐related cancers are hepatocellular carcinoma (HCC) linked to endemic Hepatitis B, C, and D; Kaposi's sarcoma (KS) caused by Human herpesvirus type 8; and Burkitt lymphoma, which is associated with the Epstein–Barr virus and malaria, particularly in children.

**FIGURE 3:**
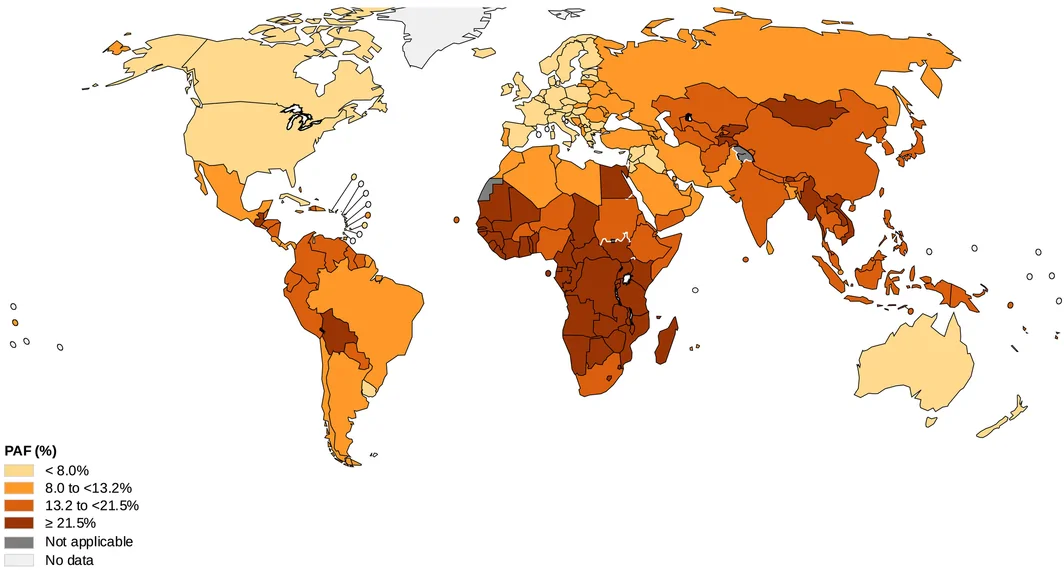
Proportion of cancers (GLOBOCAN 2020) attributable to infections. PAF, population attributable fractions.

Additionally, stomach cancer is linked to *Helicobacter pylori* and schistosomiasis‐related bladder cancer in some parts of North Africa [24]. Human immunodeficiency virus (HIV) co‐infection is driving a significant proportion of incident cervical and KS, with an estimated 70% of all HIV‐attributable cancers in 2022 occurring in SSA [25]. In terms of cancer mortality, cervical cancer and HCC—which are two of the top four causes of cancer death in Africa—are linked to infections. With scarce data on cancer etiological factors, other cancers driven by infections are yet to be identified.

### 2.3 Environmental Exposures

#### 2.3.1 Food‐Borne Carcinogens and Agricultural Exposures

Analyses of African diets revealed widespread exposure to several established human carcinogens, as classified by the IARC Monographs Program on the Identification of Carcinogenic Hazards to Humans [26]. These include polycyclic aromatic hydrocarbons (PAHs) in smoked fish and edible oils, and a fungal metabolite, aflatoxin B1 [27], a cause of HCC [28, 29]. This is particularly high in West Africa, where exposure to aflatoxin particularly in grain staples is high [30, 31] driven by modifiable sub‐optimal post‐harvest handling practices related to poverty, fear of food loss, and low education.

Other food‐borne carcinogens relate to a rise in unregulated import, sale, remixing and distribution of pesticide cocktails in SSA, often handled without personal protective equipment [32]. While carcinogenicity of many pesticides is yet to be established, the organochloride lindane, banned in 52 countries but widely used in SSA [33], is an established carcinogen linked to NHL. Moreover, studies in SSA have reported pesticide residues in human breastmilk, blood, food, water, sediment and air [34].

#### 2.3.2 Household and Urban Emissions

Household air pollution is the leading cause of premature mortality attributed to air pollution in SSA, contributing more than a third of a million deaths attributed to air pollution in 2021 [35]. Most countries affected by indoor air pollution are found in SSA, with a 1000‐fold difference in attributable death rates compared to Europe, North America, LAC, and China [36]. The major carcinogenic pollutant is coal used for household warming, mainly in Southern Africa [37]. In 2023, an estimated 80% of the population in most SSA countries relied on solid fuels for cooking, compared to barely 20% in other regions worldwide [38]. Use of solid fuels increases exposure to carcinogenic PAHs through smoke inhalation, dietary intake of solid fuel‐cooked foods and beverages, and dermal contact. Furthermore, in rapidly urbanizing areas, outdoor air pollution from diesel exhaust, unregulated industries, and open waste burning compounds household exposures, further elevating cancer risks.

#### 2.3.3 Occupational Exposures

Occupational exposures contribute to a substantial burden of cancer mortality globally. Recent estimates suggest that 0.4% of cancers in SSA are attributed to occupational exposures [12], but data were limited as occupational cancer is under‐researched in SSA. However, specific occupational exposures are likely higher in Africa compared to HIC due to limited regulation and workers' protection, informal sector activities, use of obsolete technologies and limited awareness of hazards [39]. In recent decades, mining manufacturing, and service industries like transportation have expanded. There are an estimated 9 million artisanal and small‐scale mining operators and about 54 million dependents of these activities in Africa [40] that are routinely exposed to carcinogens such as silica dust, asbestos, arsenic, beryllium, cadmium, chromium VI, and nickel. These agents are associated with increased risks of lung cancer, mesothelioma, ovarian cancer, bladder cancer, and non‐melanoma skin cancer [26]. The global chemical manufacturing industry growth rate is currently 3.5% annually and projected to double by 2030 of which two thirds is in low‐middle income countries including those in SSA [41]. The manufacturing industry is a major source of occupational exposure to carcinogenic chromium VI, PAHs, and formaldehyde.

Besides mining, the transport industry is the second source of exposure to diesel engine exhaust (DEE), which is a widespread carcinogen associated with lung cancer [42]. The United Nations Environmental Programme estimated that 40% of exported used vehicles in 2015–2018 ended up in SSA, most of which were more than 15 years old and fell below EU emission standards. They are outdated and rely on obsolete technologies and consequently higher emissions of exhaust fumes [43]. Together, these occupational exposures represent an eminent cancer risk in SSA, which is likely underestimated at present. Estimates from the GBD indicate that asbestos, silica dust, second‐hand smoke and DEE accounted for 84% of occupational carcinogen‐attributable deaths in 2016 in SSA, close to the global average. Whereas absolute deaths attributed to occupational carcinogens remain lower than Asia and LAC, trends indicate one of the highest increases, suggesting a growing occupational risk [44].

### 2.4 Cultural Factors and Local Knowledge

Conventional scientific evidence of cancer often clashes with traditional health beliefs in SSA. Knowledge on causes of cancer in some African patient populations is mixed, with more than one in three attributing cancers to supernatural causes, and only 50% attributing it to biomedical causes [45]. Hence, cancer patients often first seek care from traditional healers and religious leaders [45]. Cancer terminology often conveys fatalism, fear, tragedy, and incurability. This discourages disclosure, potentially delaying care‐seeking [46]. Similarly, previous work on the impact of cancer on families highlights how cancer extends beyond the individual to disrupt caregiving, burden households financially and emotionally, and affect children and future generations [47]. These insights underscore that cancer prevention messages should not be framed solely around biomedical outcomes but around collective social benefits, family protection, preserving livelihoods, and safeguarding community wellbeing.

The above sociocultural context would not be dismissed as a simple barrier to cancer prevention but integrated in the AfCAC, according to the IARC methodology. As included in the WCACF governance of each Regional Code [17], local knowledge would be included in different stages of the development of an AfCAC: (1) in the *preparation phase*, including formative research, for example, as a broad consultation with traditional healers, religious leaders, social anthropologists, village health teams, and community health workers, as well as policymakers to explore cancer prevention implementation priorities and determinants, and the acceptability of adopting and implementing the AfCAC in their NCCPs; (2) in the *development phase*, by ensuring a diverse selection of experts, in particular in the Communication and Health literacy Working Group, to guarantee that the AfCAC recommendations will integrate indigenous knowledge and resonate with cultural values; and (3) in the *dissemination phase*, where the Advocacy Group including representatives of influential institutions from the region (e.g., cancer leagues, medical societies, international organizations) will promote and disseminate the AfCAC supported by the respective regional and national health‐decision makers, ensuring that the recommendations are perceived as a collective good rather than an individual responsibility.

### 2.5 Healthcare Infrastructural Challenges

The scale of cancer control infrastructural deficits in SSA remains large. Primary deficits include limited cancer diagnostic and treatment facilities, limited access to effective cancer medicines, and concentration of modern oncology facilities in hard‐to‐reach urban areas. In addition, there is shortage of a robust workforce and infrastructure for cancer registration and surveillance. Even where physical infrastructure exists, the out‐of‐pocket expenditure on oncology services is high, and essential technology like radiotherapy remains sparse and frequently incapacitated by power instability or poor maintenance [15]. The development of AfCAC recommendations would acknowledge that in SSA, primary, and secondary cancer prevention strategies are still developing, and existing systemic healthcare infrastructural challenges often impede successful delivery of recommendations related to preventive interventions such as vaccination, population‐based screening and early diagnosis, and treatment and palliative care programs. Nonetheless, it is worth highlighting that cancer control infrastructure maturity differs between countries in SSA [48]. Thus, implementation of AfCAC recommendations will require progressive, stratified delivery pathways, prioritizing high‐impact, low‐cost population‐wide interventions in low‐resource settings, and more resource‐intensive interventions in high‐income countries. This can be achieved through integrating AfCAC recommendations into existing NCCPs and non‐communicable diseases plans (NCDP) to ensure alignment with national priorities, resource envelopes and implementation pathways.

## 3 Development and Implementation Governance for AFCAC

The AfCAC will be a manifestation of SSA's potential to unify scientific and community efforts against a common public health threat. Its development will be guided by the above described WCACF. This framework is a multi‐stakeholder initiative, led by IARC/WHO to promote cancer prevention globally through the development and dissemination of Regional Codes Against Cancer under a common, rigorous methodology and established governance and processes [17]. The goal is to translate the latest and best available scientific knowledge on cancer risk factors and effective preventive interventions into evidence‐based, actionable recommendations for the public in a specific region, complemented by population‐level recommendations for policy makers. This is achieved particularly in the planning, development, and dissemination phases described in the WCACF, where (1) key cancer prevention policy makers and program managers are consulted during formative research to understand cancer prevention priorities in the region in the context of existing infrastructure, (2) recommendations for the general public will be developed following the criteria 1–3 described in the WCACF methodology [17], followed by a thorough assessment of international and regional policies and a prioritization exercise, as described in criterion 4, and (3) as part of the dissemination phase, the approved AfCAC would be endorsed by the key national cancer and public health institutions in SSA, and implemented according to a dissemination and implementation strategy, jointly designed with these institutions and supporting advocacy groups during the precedent phases of the development of AfCAC.

### 3.1 Afrocentric Ownership of the AfCAC

The AfCAC would be a Pan‐African initiative developed together by African partners and coordinated by the IARC to provide the evidence assessment methodology and governance as described in the WCACF. In this case, the IARC would liaise with the strong organizational leadership of cancer‐dedicated and public health institutions in Africa as they appoint scientific experts. Established organizations such as the WHO Regional Office for Africa (AFRO), the Africa Centre for Disease Control and Prevention (AfCDC), and African Organisation for Research and Training in Cancer (AORTIC) are potential strategic partners with expertise in disease prevention required to shape AfCAC development in leadership, scientific and technical capacities. WHO AFRO has a regional strategy for cancer prevention and control, as well as a regional coordination strategic framework to effectively combat cancer. WHO AFRO's established mandate, regional authority, and extensive network across all member states, and its track record of actively implementing and supporting major regional cancer prevention initiatives demonstrate potential leadership of AfCAC development. The AfCDC has a mandate to implement a continental strategy for prevention and control of NCDs including cancer. The AORTIC, now IARC's official partner, in collaboration with leading European institutions, leads capacity training programs such as African cancer leadership institute which is dedicated to training young African scientists in cancer epidemiology and research leadership. Accordingly, the immense human resource capacity necessary to constitute the several scientific Working Groups and Committees required to deliver on this ambitious project cannot be underestimated and needs to be ignited now.

### 3.2 Evidence Base

Scientific rigor and conformity of cancer prevention recommendations are the core principles of the WCACF and methodology. Importantly, the culture and religious beliefs and practices would be consulted and incorporated accordingly into cancer prevention message framing under the expert advice of the Communication and Health Literacy WG. As explained above, attention will need to be paid to how these barriers can be transformed into positive cancer prevention messages through broad consultation with cultural and religious leaders, traditional healers, social anthropologists, village health teams, and cancer advocacy groups with adequate understanding of community needs and barriers to cancer prevention, and who are usually the initial points of contact for cancer patients. This will ensure that while firmly rooted in scientific evidence, the AfCAC also integrates African knowledge, resonates with the continent's diverse cultures and local experiences, and is a collective output of African wisdom, hence building community trust, acceptance, and successful uptake.

### 3.3 Funding

The success of this ambitious and comprehensive project requires intensive time and financial investment. For example, the development of the fifth edition of the ECAC involved 80 European experts in different capacities and 13 leading national institutions endorsed the recommendations and supporting outputs and costed more than 1,500,000 Euros [49]. Therefore, ability of African stakeholders to mobilize financial support from national governments, corporate social responsibility initiatives, philanthropic organizations as well as international donors will be key to the development of the AfCAC. Accordingly, we call upon the African Union (AU), reflecting on the health aspiration of the AU Agenda 2063 [50], to plan and allocate funds for the AfCAC during the next 10‐year implementation plans. We note that cancer prevention and control have not explicitly been highlighted in the AU Agenda 2063 but believe that investing in the AfCAC will mitigate the current cancer trends, offer generous health and economic returns to member states, and will be fundamental in achieving the AU transformation Agenda—“the Africa we want.”

Limited financial support will need to be complemented by mobilization of domestic human resources, such as innovative and voluntary contributions from scientists who are keen on contributing to African science. This financing modality was echoed in 2017 in the 70th World Health Assembly (WHA70.12) agenda 15.6—the cancer prevention resolution. Besides, communal effort is traditionally a powerful asset in many African societies, and this should be leveraged for public health good, in line with Aspiration #6 of the AU Agenda 2063: “An Africa whose development is people‐driven, relying on the potential of the African People.” Young African scientists currently benefiting from cancer research capacity building programs globally are a rich resource that should be mobilized to voluntarily contribute to evidence synthesis for the AfCAC.

### 3.4 Dissemination and Implementation

For the AfCAC to achieve its intended impact, dissemination must go beyond publication in scientific or policy documents into formats that speak to everyday life. Africa's linguistic diversity requires deliberate translation into major African languages and adaptation into culturally relevant formats, with dissemination pursued at national and community levels. At the national level, mass media such as radio and television remain powerful tools for wide reach, and the policy recommendations should be implemented across Ministries and national institutions. Integration into NCCPs, school curricula, and workplace health programs will help institutionalize the Code.

Regional bodies such as the AU, WHO‐AFRO, AfCDC, and AORTIC can coordinate and harmonize efforts across borders. At the community level, diverse dissemination strategies in the local languages, including theatre, music, and storytelling may provide trusted avenues for engagement. Religious and traditional institutions, alongside communal markets, schools, and workplaces, are well placed to deliver messages that resonate with people's daily lives. At the digital level, mobile health platforms, SMS reminders, and WhatsApp groups can deliver simple prevention tips directly to households. Social media campaigns, infographics, comics, and even radio or TV dramas can engage younger populations and ensure accessibility across all literacy levels. Across all levels, messaging should emphasize shared social benefits—protecting families, safeguarding livelihoods, and strengthening community wellbeing—while promoting hope and collective resilience rather than fear and fatalism.

## 4 Conclusions

Recognizing SSA's unique cancer context, the adverse health and economic impacts associated with an increasing cancer burden, and constrained healthcare infrastructure, we argue that a dedicated Afro‐centric set of evidence‐based cancer prevention recommendations is long overdue. Great strides have been made to establish cancer prevention programs, including NCCPs and NCD plans which incorporate cancer prevention which have been developed by 81% of countries in Africa [14]. However, limited participation in cancer prevention initiatives in Africa, in some cases reported at 10%–50%, reflects fragmented programming, inadequate population outreach, low awareness, health illiteracy, vaccine hesitancy, stigma, and absence of age‐ and gender‐responsive services [15]. An AfCAC would represent authoritative, more systematic, continent‐wide, evidence‐based guidance that addresses Africa's specific epidemiological and socio‐cultural contexts. NCCPs remain a foundational authoritative source of local knowledge about cancer control and prevention that can supported and updated by AfCAC recommendations. We anticipate that implementation of the AfCAC will be cost‐effective, save lives, increase productivity, and ultimately contribute to the Pan‐African goal of inclusive and sustainable development, while also signaling African leadership in setting its own cancer prevention agenda.

## Author Contributions

**Anthony Kityo:** conceptualization, writing – original draft, writing – review and editing. **Phyllis Ohene‐Agyei:** conceptualization, writing – original draft, writing – review and editing. **Felix M. Onyije:** writing – original draft. **Hannah Simba:** writing – original draft. **Annet Nakaganda:** writing – review and editing. **Denis Chopera:** writing – review and editing. **Kofi Mensah Nyarko:** writing – review and editing. **Benido Impouma:** writing – review and editing. **Mary Nyangasi:** writing – review and editing. **Blandina Mmbaga:** writing – review and editing. **Valerie McCormack:** writing – review and editing, validation, supervision, resources. **Cesaltina Lorenzoni:** writing – review and editing. **Miriam Mutebi:** writing – review and editing. **Joachim Schüz:** writing – review and editing, validation, project administration, resources, supervision, methodology. **Carolina Espina:** writing – review and editing, validation, methodology, supervision.

## Disclosure

Where authors are identified as personnel of the International Agency for Research on Cancer/World Health Organization, the authors alone are responsible for the views expressed in this article and they do not necessarily represent the decisions, policy or views of these organizations.

## Conflicts of Interest

The authors declare no conflicts of interest.
